# The perception of COVID-19 among Italian dental patients: an orthodontic point of view

**DOI:** 10.1186/s40510-021-00355-7

**Published:** 2021-04-07

**Authors:** Stefano Martina, Alessandra Amato, Paolo Faccioni, Alfredo Iandolo, Massimo Amato, Roberto Rongo

**Affiliations:** 1 Department of Medicine, Surgery and Dentistry “Scuola Medica Salernitana”, University of Salerno, Via Allende, 84081 Baronissi, SA Italy; 2 Department of Surgery, Dentistry, Paediatrics and Gynaecology, University of Verona, Policlinico G.B. Rossi - P.le L.A. Scuro 10, 37134 Verona, Italy; 3 Department of Neurosciences, Reproductive Sciences and Oral Sciences, University of Naples Federico II, Via Pansini 5, 80131 Naples, Italy

**Keywords:** COVID-19, Dental practice, Dental patient, Survey, Orthodontics, TMD

## Abstract

**Background:**

The present study aimed to investigate the patients’ perception of the dental practice during the COVID-19 outbreak, and whether the pandemic will affect the attendance of orthodontic patients at the dental practice. An online questionnaire, including the Patient Health Questionnaire-4 (PHQ-4), was submitted to Italian dental patients with items about their perceived risks when going to the dentist, concerns about continuing orthodontic treatment, and the onset of temporomandibular disorders (TMD). Data were analyzed with a chi-square test and logistic regression analysis. The level of significance was set at *P* < 0.05.

**Results:**

A total of 1566 subjects completed the survey, including 486 who were under orthodontic treatment or who had a child in orthodontic treatment. A total of 866 participants (55.3%) thought the risk of contracting the COVID-19 infection was higher in a dental practice; this perception was associated with gender (women more than man), age (over 60 years old) and high levels of distress (*P*<0.001). However, 894 patients (57.1%) felt comfortable going back to the dentist. Most of the orthodontic patients (84%) would continue their treatment. After the lockdown, there was a slight increase in the frequency of TMD pain (356 versus 334).

**Conclusions:**

Most of the participants believed that the dental practice is a place at greater risk of contracting COVID-19, even if they continue to go to the dentist. Gender, age, and the level of distress were associated with the increase in the fear of going to the dentist due to COVID-19. Because of the pandemic, 16% of patients undergoing orthodontic treatment would not return to the dental practice to continue their orthodontic treatment after the lockdown. The prevalence of TMD pain in the population increased due to the pandemic.

## Background

An epidemic caused by a novel coronavirus (SARS-COV-2) broke out in China in December 2019 and spread worldwide in the following months [1]. The disease caused by this virus was named corona virus disease 2019 (COVID-19), and in March 2020, a pandemic was declared by the World Health Organization [2]. The clinical spectrum of COVID-19 is wide, from asymptomatic cases and mild symptoms like sore throats, headaches, and nasal congestion to more severe clinical pictures such as fevers, coughs, dyspnoea, myalgia, severe viral pneumonia with respiratory failure, and even death [3]. Although they were not initially evidenced, olfactory and gustatory disorders are also very common symptoms in COVID-19 patients [4]. The patients at a greater risk of severe disease with a high probability of hospitalization in the intensive care unit are those with comorbidities such as chronic obstructive pulmonary disease, cardiovascular disease, diabetes, and hypertension [5]. Besides, it was found that COVID-19 could cause cardiovascular injuries such as pericarditis, myocarditis, myocardial infarction, heart failure, arrhythmias, or thromboembolic events [6]. COVID-19 has a high transmissibility rate (higher than that of SARS) [7] because a high number of COVID-19-positive patients has mild or no symptoms, which makes it difficult to diagnose, so the spread of infection can occur at an accelerated rate [8]. In the absence of effective vaccines or treatments, the only available method to reduce SARS-CoV-2 transmission as much as possible is by identifying and isolating infected patients who are contagious and can transmit the disease [9]. The nasopharyngeal RT-PCR swab tests are currently the gold standard for diagnosing COVID-19. The test has high sensitivity but moderate specificity, a long processing time, high costs, and its accuracy depends on the quality of the samples provided [10]. This often can open a gaping hole in SARS-CoV-2 prevention efforts.

SARS-COV-2 can propagate between people through coughing, sneezing, and the inhalation of virus-containing droplets and micro-droplets from infected individuals [11]. Several studies demonstrated that the transmission of COVID-19 may occur with aerosols generated during dental procedures [12]. Moreover, in the absence of any disinfection procedure, SARS-CoV-2 has a half-life of 6.8 h on plastic surfaces and 5.6 h on stainless steel surfaces [13]. In this scenario, it is obvious that a dental practice can be considered a high-risk infection place. In March, Italy adopted quarantine measures to control the spread of COVID-19 and it was recommended that dentists interrupt their activities and provide treatments for dental emergencies only. In May, with the publication of guidelines by the Italian Ministry of Health [14], the normal activity of dental practices resumed. Nevertheless, most Italian dentists were afraid to return to their daily activities, considering their job a high COVID-19 risk for them and their families [15]. Since September 2020, Italy and European countries faced a second wave of the pandemic, with more cases than the first wave [16]. However, in this last period, different from the spring lockdown, the activity of dental practices did not stop, but it is difficult to determine whether patients feel that returning to the dentist during the pandemic wave is safe. Indeed, several studies have investigated the impact of COVID-19 on the dental profession, but only two have been carried out from the patient’s point of view. Both were conducted in Brazil [17, 18].

Among the different dental branches, orthodontics is the one that produces aerosols less frequently compared with others, such as restorative dentistry, endodontics, or prosthodontics. However, orthodontics mainly treats children, who were indicated very frequently as asymptomatic carriers of the virus [19]. Hence, orthodontic patients or parents of orthodontic patients may consider a return to the dental practice an increased risk to their healthy status.

The aim of this study was to investigate the perception of the dental practice among people after the lockdown period, and if the pandemic affected the attendance of orthodontic patients to the dental practice.

## Methods

**Fig. 1:**
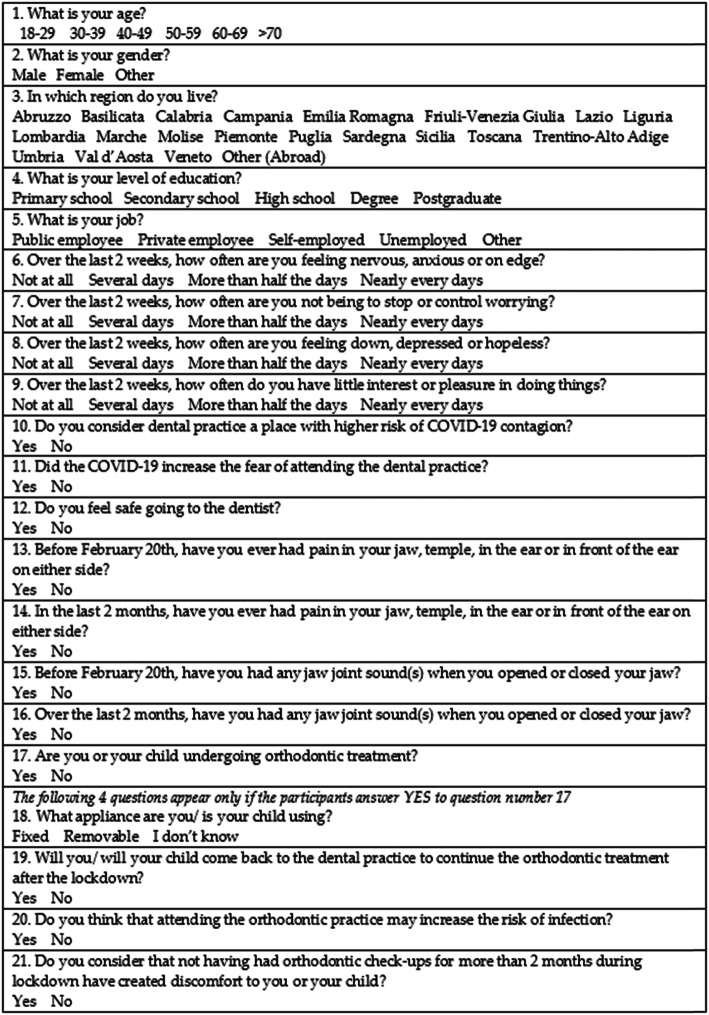
Online survey questionnaire (translated in English)

The present cross-sectional study was conducted by sending an online survey questionnaire (Fig. 1) to persons who have sought dental cares during this year. The survey was created with an online survey development cloud-based software called SurveyMonkey® (SVMK, San Mateo, CA, USA). The participants were approached via Facebook, WhatsApp, and mailing lists. Participants authorized the treatment of their data, which was collected anonymously. The questionnaire was comprised of 21 multiple-choice questions: 5 questions were about personal data (gender, age, living region, level of education, profession), 3 were about the anxiety over going to the dentist, 4 were about the presence of TMD symptoms, and 5 were about orthodontic treatment and the perception of the risk of continuing orthodontic treatment. The Patient Health Questionnaire-4 [20] was included in the survey to assess whether the general state of anxiety and depression might be associated with the other answers. The PHQ-4 was composed of four questions with a 4-item Likert scale. Scores between 3 and 5 indicated slight distress, between 6 and 8 indicated moderate psychological distress, and higher than 10, a severe distress. In the survey, most of the questions had a dichotomous answer (yes/no).

### Statistical analysis

Frequencies and percentages for categorical data were computed. A chi-square test was used to assess the association between gender (male vs. female), age (older than 40 years old vs. 18–39), and level of distress (0–1 vs. 2–3). In case of a statistically significant association, logistic regression analyses to calculate the odds ratio (OR) were performed. A standard statistical software package (SPSS, version 22.0; SPSS IBM, Armonk, New York, USA) was used. The level of significance was set at *P* < 0.05.

## Results

A total of 1566 patients (698 M, 852 F, 16 NR, Table 1) completed the survey. Participants were asked to identify their age group, with those between 18 and 39 years old being the most common (956). Most patients come from southern regions of Italy (1142). Most participants had a high school (650) or higher education level (544) and were self-employed (412).

**Table 1:** General data of the participants

	Frequency (percentage)					
**Age**	18–29 490 (31.3%)	30–39 466 (29.8%)	40–49 286 (18.3%)	50–59 166 (10.6%)	60–69 106 (6.8%)	>70 46 (3%)
**Gender**	Males 708 (45.2%)			Females 858 (54.8%)		
**Living region**	Centre-North 424 (27.1%)			South 1142 (72.9%)		
**Level of education**	Secondary school 76 (4.8%)	High school 650 (41.5%)		Degree 544 (34.7%)	Postgraduate 292 (18.6%)	
**Profession**	Public employee 202 (12.9%)	Private employee 264 (16.9%)	Self employed 412 (26.3%)	Unemployed 216 (13.8%)	Other 472 (30.1%)	
**PHQ-4**	No/low distress 1226 (78.3%)			Moderate/high distress 336 (21.7%)		

A total of 866 patients (55.3%) believed that there is a greater risk of contracting COVID-19 at a dental practice, but 894 patients (57.1%) felt comfortable going back to the dentist (Table 2). Men were less afraid to go to a dental clinic; indeed, among those who were less afraid, 450 (52.8%) were men, and 402 (47.2%) were women, while among 714 who were more afraid, 258 (36.1%) were men, and 456 (63.9%) were women (*P*<0.001). Younger people were less scared to go to a dental clinic in comparison with people over 40 (*P*<0.001).

**Table 2:** Frequencies and percentages of replies to perceived risk and fear about attending dental practice

	Frequency (percentage)	
**Consider dental practice at higher risk**	Yes 866 (55.3%)	No 700 (44.7%)
**COVID-19 increased the fear of attending the dental practice**	Yes 714 (45.6%)	No 852 (54.4%)
**Feel safe going to the dentist**	Yes 894 (57.1%)	No 672 (42.9%)

The PHQ-4 reported 1226 participants with no/low distress and 336 participants with moderate/high distress (>6); 4 participants did not complete the PHQ-4 (Table 1). Higher levels of distress, anxiety, and depression were found in women (*P*<0.001) and young people (<40 years old, *P*=0.04). A high PHQ-4 score was associated with a fear of going to a dental practice; indeed, 55.4% (186/336) of the subjects with higher distress levels were more fearful of going back to the dentist compared with 42.9% (526/1226) of the subjects with less distress (*P*<0.001).

Among the 486 participants who were in orthodontic treatment or who had a child in orthodontic treatment, 408 (84.0%) responded that they would continue the treatment, but 232 (47.7%) thought that going to the dental practice for orthodontic appointments may increase the risk of infection. Of the 78 patients who will not return to the dental practice after the lockdown, 62 (79.5%) thought that in the dental practice there was a greater risk of contracting COVID-19, and for 66 (84.6%) COVID-19 increased their fear of going to the dentist (Tables 3 and 4). Among the 272 subjects who were in orthodontic treatment and consider the risk of COVID-19 transmission in dental practices to be higher, 194 (71.3%) thought that going for an orthodontic control increased their risk of being infected, and for 170 (70.8%), the pandemic increased their fear of going to the dentist. Among the 263 patients with orthodontic fixed treatment, 243 (92.4%) will return to the dental practice, while among 174 patients with removable appliances, only 142 (81.6%) declared that they would continue their treatment. In addition, 218 patients (44.9%) reported that not having been monitored by their orthodontist for more than 2 months caused them discomfort. Among those, 76 (34.9%) were treated with removable devices, 120 (55%) with fixed appliances (*P*=0.01), and the other 22 (10.1%) did not know the type of appliance.

**Table 3:** Frequencies and percentages of replies to questions about orthodontics

	Frequency (percentage)		
**In orthodontic treatment or have a child in orthodontic treatment**	Yes 486 (31.0%)	No 1080 (69.0%)	
**Characteristics of orthodontic appliance**	Fixed 249 (51.2%)	Removable 174 (35.8%)	Don’t know 63 (13%)
**Back to the dental practice to continue the orthodontic treatment after lockdown**	Yes 408 (84.0%)	No 78 (16.0%)	
**Think that attending the orthodontic practice may increase the risk of infection**	Yes 232 (47.7%)	No 254 (52.3%)	
**Consider that not having had orthodontic check-ups during lockdown created discomfort**	Yes 218 (44.9%)	No 268 (55.1%)

**Table 4:** Chi-square test and regression analysis for question related to the orthodontic treatment, and gender, age, and PHQ-4 (sample 486)

	Gender		Age		PHQ-4	
	Χ^2^	OR (95% CI)	Χ^2^	OR (95% CI)	Χ^2^	OR (95% CI)
**Back to the dental practice to continue the orthodontic treatment after lockdown**	0.132		0.059		0.576	
**Think that attending the orthodontic practice may increase the risk of infection**	0.475		0.059		**0.004**	**1.83 (1.21-2.75)**
**Consider that not having had orthodontic check-ups during lockdown created discomfort**	0.761		0.096		**0.011**	**1.69 (1.12-2.54)**

*Χ*^2^ *P* value of the chi-squared test, *OR* odds ratio of the logistic regression model, *95% CI* 95% confidence interval of the odds ratio

Regarding TMD, 334 (21.3%) patients referred to having TMD-related pain before the start of the epidemic and 356 (22.8%) reported pain during the lockdown (Table 5). In both groups, the pain was more frequent in women (63.5% and 65.2% respectively; *P<*0.001), while there was no association with age. Similarly, 336 (21.5%) patients reported TMJ sounds before February 2020, whereas 304 (19.4%) mentioned TMJ sounds during the months of the lockdown, with a significant prevalence in women (188/304, *P*=0.03) and people under 40 (210/304, *P*=0.03) only in the second group. Interestingly, there was an association between high levels of distress and the presence of pain (*P*=0.006) or TMJ sounds (*P*=0.004) after the onset of the pandemic (Table 6).

**Table 5:** Frequencies and percentages of replies to questions about temporomandibular disorders

	Frequency (percentage)	
**Pain in your jaw, temple, in the ear or in front of the ear on either side before the pandemic**	Yes 334 (21.3%)	No 1232 (78.7%)
**Pain in your jaw, temple, in the ear or in front of the ear on either side during the lockdown**	Yes 356 (22.8%)	No 1210 (77.2%)
**Jaw joint sound(s) when you opened or closed your jaw, before the pandemic**	Yes 336 (21.5%)	No 1230 (78.5%)
**Jaw joint sound(s) when you opened or closed your jaw, during the lockdown**	Yes 304 (19.4%)	No 1262 (80.6%)

**Table 6:** Chi-square test and regression analysis for question related to anxiety toward attending the dental practice, presence of TMD symptoms, and gender, age, and PHQ-4

	Gender	Age		PHQ-4		
	Χ^2^	OR (95% CI)	Χ^2^	OR (95% CI)	Χ^2^	OR (95% CI)
**Consider dental practice at higher risk**	0.573		0.418		0.790	
**COVID-19 increased the fear of attending the dental practice**	**<0.001**	**1.98 (1.61–2.41)**	**<0.001**	**0.63 (0.51–0.77)**	**<0.001**	**1.65 (1.29–2.1)**
**Feel safe going to the dentist**	**<0.001**	**0.67 (0.55–0.82)**	**0.018**	**1.28 (1.04–1.57)**	**<0.001**	**0.59 (0.47–0.76)**
**Pain in your jaw, temple, in the ear or in front of the ear on either side before the pandemic**	**<0.001**	**1.58 (1.23–2.02)**	**0.112**		**0.029**	**1.37 (1.03–1.82)**
**Pain in your jaw, temple, in the ear or in front of the ear on either side during the lockdown**	**<0.001**	**1.74 (1.37–2.23)**	0.062		**0.002**	**1.54 (1.18–2.03)**
**Jaw joint sound(s) when you opened or closed your jaw, before the pandemic**	0.141		<0.001	1.69 (1.3**–**2.2)	0.008	1.46 (10**–**1.93)
**Jaw joint sound(s) when you opened or closed your jaw, during the lockdown**	**0.006**	**1.43 (1.11–1.85)**	**0.001**	**1.57 (1.2–2.05)**	**0.004**	**1.52 (1.14–2.03)**

*Χ*^2^ *P*value of the chi-squared test, *OR* odds ratio of the logistic regression model, *95% CI* 95% confidence interval of the odds ratio

## Discussion

This study was carried out immediately after the end of the lockdown in Italy (May 2020). The aim was to understand through a survey how concerned dental patients were about the possibility of contracting an infection in the dental practice and if this may affect their attendance at the dentist and the orthodontist.

The sample was a good representation of different age ranges, different levels of education, different jobs, and different levels of distress. The PHQ-4 indicated that 336 participants (21.7%) had moderate/severe psychological distress (score equal to or higher than 6) during the lockdown period. This percentage was greater compared with the data presented of the general population in the pre-pandemic period, where about 5% of the population presented these scores [21]. It could be assumed that the tough situation of the pandemic might influence the psychological status of the participants.

Almost half of the patients (44.7%) thought that the dental practice is a place where there is a higher risk of COVID-19 infection; in 45.6% of participants, COVID-19 increased their fear of attending the dental practice, and 42.8% did not feel safe to go back to the dentist. Among these, the most scared were older people (> 60 years old) and women. Regarding the elderly, it is quite obvious that they were more scared because COVID-19 affects them more frequently and more severely [22]. The association with women is consistent with the data from a previous study by Cotrin et al., which found a greater level of anxiety among female patients compared to males [18]. Moreover, these results are in accordance with our findings of the PHQ-4, which indicated higher levels of distress, anxiety, and depression in women, and with a nationwide survey of psychological distress among Chinese people in the COVID-19 epidemic, where women showed significantly higher psychological distress than men [23]. It must be considered that in dental practices, there were already strict protocols for infection control of several infective diseases, such as hepatitis B, hepatitis C, human immunodeficiency virus infection, tuberculosis, and herpes simplex, even before the COVID-19 pandemic [24]; therefore, dentists have always been prepared to face viruses. With the COVID-19 pandemic, these protocols became even more stringent [25] and in May 2020 the minister of health published national guidelines [14]. Furthermore, it was observed that more change in behaviors was associated with lower country COVID-19 fatality rates [26]. For these reasons, dentists should be able to improve communication with their patients, considering that some patients’ characteristics could induce fear of returning to the practice, and calm those who are more anxious. Indeed, patients not returning to the dental practice could be considered an indirect harmful effect of the COVID-19 pandemic, especially those who must be treated with frequent controls, such as patients with periodontal disease or those undergoing orthodontic treatment.

A part of the questionnaire was dedicated to patients in orthodontic treatment or parents of patients in orthodontic treatment. Sixteen per cent of respondents declared that they would not return to the dental practice to continue orthodontic treatment. Among those, 83.8% thought the dental practice was a place with greater risk of contracting COVID-19 and 89.2% were scared to go to the dentist due to the pandemic. These findings are consistent with previous studies, showing that patients who were willing to go to the dental office were more calm or indifferent, and reporting significantly lower scores of anxiety than patients that would not go [17, 18]. The dentist should explain to the more anxious patients that the practice is a safe place and that the damage resulting from a missed control of the orthodontic appliance could be serious. It should also be noted that orthodontic procedures have a low risk of infection because most of them do not require aerosols. Therefore, correct communication between patient and orthodontist is of fundamental importance. Meanwhile, orthodontists should re-organize their schedules for the following reasons: to reduce the number of patients per day, to prevent gatherings in the waiting room, and to perform the disinfection procedures provided in the guidelines. In addition, 41.8% of patients reported that not having been monitored by the orthodontist for more than 2 months caused them discomfort; those with fixed appliances in particular were more disturbed. This could be explained by the fact that removable appliances generally cause less discomfort to patients and result in a lower average number of emergency visits as compared with fixed appliances [27, 28]. Hence, it could be argued that for this reason the COVID-19 pandemic could accelerate the trend already occurring in orthodontics towards an increase in removable appliances, such as clear aligners or activators.

The survey also had four questions to investigate the presence of TMD before the onset of the pandemic and after the lockdown. The 21.3% of respondents referred to having TMD-related pain and 21.5% reported TMJ sounds before the start of the pandemic. These data were consistent with the prevalence of TMD reported in previous Italian epidemiological studies [29, 30]. The pain and the TMJ sounds were more frequent in women, according to current scientific evidence [31]. After the lockdown, there was a slight increase in the frequency of pain, while TMJ sounds decreased. In addition, there was an association between high levels of distress and the presence of pain or TMJ sounds after the outbreak of COVID-19. This could be due to the relation between the onset of muscular pain or TMJ sounds and the level of distress and anxiety of subjects. In particular, during the lockdown, people presented a higher level of distress and anxiety due to the complex situation that may contribute to the prevalence of TMD pain.

This study presents some limitations: it is a survey-based study; thus, information is self-reported, and the collection time was only 2 weeks. However, it also presents some strengths, such as the high number of participants, the good representation of the population, and its depiction of the situation of Italian dental patients, focusing on those with TMD or under orthodontic treatment.

## Conclusions

This survey-based study investigated the risk perception of Italian patients toward attending dental practices during the COVID-19 pandemic.

Most of the participants believed that dental practices are places at greater risk of COVID-19 transmission, even if they will continue to go to the dentist. Gender, age, and the level of distress were associated with the increase in the fear of attending dental practices due to COVID-19.

Because of the pandemic, 16% of patients undergoing orthodontic treatment would not return to the dental practice to continue their orthodontic treatment after the lockdown. Patients with fixed appliances felt more discomfort in not having had orthodontic check-ups during the lockdown compared to patients with removable appliances.

The level of distress was associated with the presence of TMD, and the prevalence of TMD pain in the population increased due to the pandemic.

Taking into account the findings of this study, dentists should strive to follow the COVID-19 national guidelines and put efforts into the communication with their patients, highlighting the safety of the dental practice and the importance of continuing the therapies.

## Data Availability

Data are available on justified request to the authors.
